# Nanocasting of Periodic Mesoporous Materials as an Effective Strategy to Prepare Mixed Phases of Titania

**DOI:** 10.3390/molecules201219812

**Published:** 2015-12-08

**Authors:** Luther Mahoney, Shivatharsiny Rasalingam, Chia-Ming Wu, Ranjit T. Koodali

**Affiliations:** Department of Chemistry, University of South Dakota, 414 E. Clark Street, Vermillion, 57069 SD, USA; Luther.Mahoney@coyotes.usd.edu (L.M.); Shivatharsiny.Rasalingam@coyotes.usd.edu (S.R.); Chia-Ming.Wu@coyotes.usd.edu (C.-M.W.)

**Keywords:** nanocasting, hard-template, titanium dioxide, TiO_2_, mesoporous, periodic, hexagonal, TiO_2_(B), hydrogen production, water-splitting

## Abstract

Mesoporous titanium dioxide materials were prepared using a nanocasting technique involving silica SBA-15 as the hard-template. At an optimal loading of titanium precursor, the hexagonal periodic array of pores in SBA-15 was retained. The phases of titanium dioxide could be easily varied by the number of impregnation cycles and the nature of titanium alkoxide employed. Low number of impregnation cycles produced mixed phases of anatase and TiO_2_(B). The mesoporous TiO_2_ materials were tested for solar hydrogen production, and the material consisting of 98% anatase and 2% TiO_2_(B) exhibited the highest yield of hydrogen from the photocatalytic splitting of water. The periodicity of the pores was an important factor that influenced the photocatalytic activity. This study indicates that mixed phases of titania containing ordered array of pores can be prepared by using the nanocasting strategy.

## 1. Introduction

Titanium dioxide has been extensively studied because of its relative ease of synthesis, benign nature, and photocorrosion stability compared with other semiconductors, such as ZnO, *etc.* [1,2]. Titania has been employed in various applications, such as solar energy utilization for water splitting, as active material in dye-sensitized solar cells, for environmental remediation of pollutants, elimination of bacteria, bio-medical arenas, and electrochromic applications [1,2,3,4,5,6,7,8]. Porous titanium dioxide is favorable in applications involving mass transfer, such as photocatalysis. Many synthetic routes have been attempted for forming mesoporous titanium dioxide, such as room-temperature drying [9,10], low-temperature supercritical drying [11,12,13,14,15], high-temperature supercritical drying [16,17], hydrothermal [18,19,20], Evaporation-Induced Self-Assembly (EISA) [21,22,23], microwave [24,25], nanocasting employing soft template [26], and hard template [27,28,29,30,31,32,33,34,35,36,37,38,39,40]. The room-temperature drying of titanium dioxide gel to form a xerogel involves long evaporation time period with pores that are non-uniform in nature; therefore, this synthetic method is of little use in forming periodic array of pores [9,10]. The low-temperature supercritical drying (LT-SCD) method forms an aerogel with favorable textural properties compared to the room-temperature prepared xerogel titanium dioxide material [11,12,13,14,15]. The low-temperature supercritical drying method forms titanium dioxide of lower crystallinity in general compared to the high-temperature supercritical drying method [16,17]. However, the challenge with the high-temperature supercritical drying (HT-SCD) process is the use of expensive apparatus and issues concerning safety. In comparison to the HT-SCD method, the hydrothermal or solvothermal method could form mesoporous titanium dioxide in a relatively shorter period of time [18,19,20]. The hydrothermal synthetic method produces titanium dioxide of similar crystallinity as the high-temperature aerogel method, but the pores have random arrangement. The Evaporation-Induced Self Assembly (EISA) synthesis might be an alternative to the synthetic methods discussed thus far [21,22,23]. However, the EISA synthesis method requires removing surfactants or templates using elevated temperatures in air [41]. The result of removing surfactants at high temperatures invariably leads to aperiodic porous structures, especially when cationic surfactants, such as cetyltrimethylammonium bromide (CTAB), are used. The microwave synthesis might be favorable with its short synthesis time period, but the pore structure of titanium dioxide will be random in nature [24,25]. As discussed above, many of these methods do not necessarily lead to the formation of nanomaterials with periodic and regular arrangement of pores. This is particularly difficult when attempting to prepare titanium dioxide with periodic arrangement of pores. This is because the calcination temperature used for removal of the surfactants usually causes crystallization of titanium dioxide particles. During crystallization, the rearrangement of the crystal structure (from an amorphous form) causes restructuring and hence collapse of the pore wall. An alternative method could be the nanocasting strategy using a soft or a hard template. Therefore, the use of soft or hard-template appears to be the avenues for retaining the periodic porous structure after calcination. The major disadvantage of the soft-template using various polymers involves the added expense and care needed in retaining the periodic array of pores using various calcination temperatures [26]. Finally, the hard-template synthesis involves multiple impregnation steps of the titanium precursor with heating time periods to slowly form a periodic arrangement of titanium dioxide under ideal conditions [27,28,29,30,31,32,33,34,35,36,37,38,39,40].

Earlier reports have indicated that mixed phases of titanium dioxide exhibit higher activity compared with the pure phase, such as anatase [42,43,44]. The higher photocatalytic activities in many reactions using TiO_2_ P25 can be attributed to the mixed phases of anatase and rutile. An optimal amount of rutile crystallites on anatase TiO_2_ has been shown to increase the photocatalytic water splitting outcomes [42]. Another work also notes that having small crystallites of rutile TiO_2_ on anatase TiO_2_ favors higher photocatalytic activity from forming “hot spots” [45]. Therefore, it is highly advantageous to identify synthesis methods that produce mixed phases of titanium dioxide in a facile manner. Previous reports preparing TiO_2_ having mixed phases with surfactants required elevated calcination temperatures in the range of 600 °C to 1200 °C to form the desired phases [46]. In addition, highly reactive TiCl_4_ or combinations of TiCl_4_ with Ti(OR)_4_ were required for forming the mixed phases of titanium dioxide [47]. Hydrothermal synthesis treatment was needed to form the mixed phase TiO_2_ material under certain conditions [48]. Other synthetic methods used for forming mixed phases of titanium dioxide include microemulsion [49], flame pyrolysis [50], and physical vapor deposition [51]. The EISA synthesis has also been performed to produce mixed phases of titanium dioxide; however, the materials lack periodic porous structure [42]. These former reports provide motivation for developing a relatively simple synthetic method in producing mixed phases of titanium dioxide that has periodic porous structure using the hard-template, mesoporous silica SBA-15.

The formation of mixed phases of anatase, rutile, and TiO_2_(B) as function of loading and nature of titanium alkoxide precursor were carefully investigated using the hard-template synthetic method. The resulting mixed phase titanium dioxide materials were evaluated for solar hydrogen production. The mixed phase titanium dioxide material with periodic hexagonal phase and having anatase (98%) and TiO_2_(B) (2%) without platinum co-catalyst exhibited the highest solar hydrogen activity. The results suggest mixed phases of titanium dioxide with periodic porous structure leads to the higher solar evolution compared with previous literature works in preparing mesoporous titanium dioxide having periodic network array of pores. Therefore, the synthetic method employed in this work enables modulation of the phase(s) of titanium dioxide by simply varying the impregnation cycles and nature of the titanium alkoxide precursor. This work suggests that periodic titanium dioxide can be produced as powders having mixed phases in a simple synthetic and controlled manner.

## 2. Results and Discussion

### 2.1. Physico-Chemical Characterization

#### 2.1.1. Powder X-ray Diffraction (XRD)

**Figure 1:**
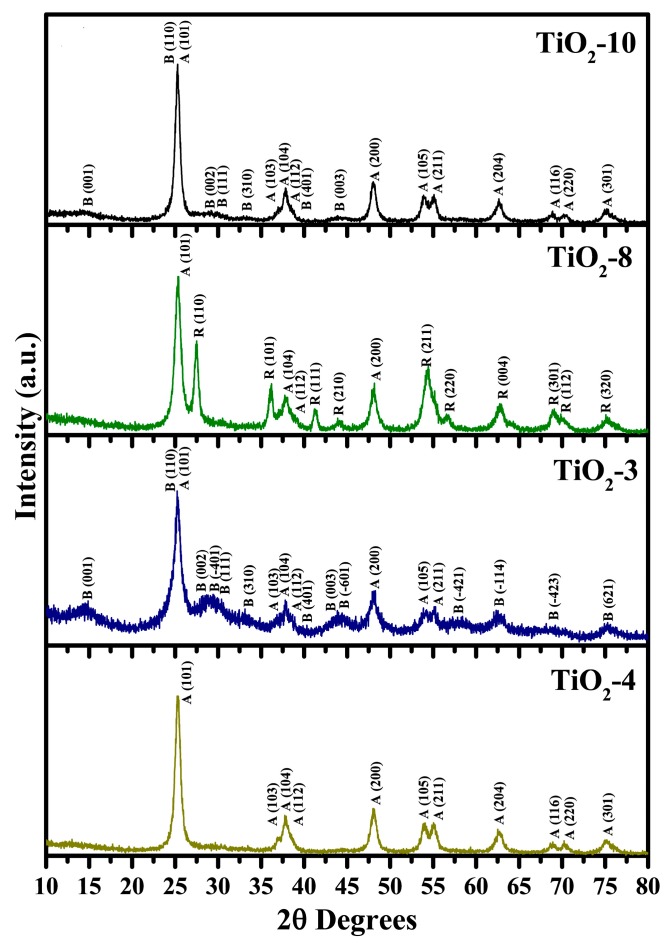
Powder X-ray diffraction of hard templated mesoporous TiO_2_ as function of impregnation cycles and titanium precursor.

A nanocasting strategy was attempted to prepare titania based nanocrystalline materials. Figure 1 shows the high-angle powder XRD data for the titania nanomaterials prepared using titanium isopropoxide and titanium ethoxide as precursors. Three of the titanium dioxide nanomaterials were made using titanium ethoxide (TiO_2_-10, TiO_2_-8, and TiO_2_-3), and the fourth material, TiO_2_-4, was prepared using titanium isopropoxide as the precursor for titania. The number after the hyphen represents the number of cycles completed in impregnating the hexagonal silica SBA-15 material in the nanocasting process. At low impregnation cycle, with the titanium ethoxide precursor, a mixed phase anatase-TiO_2_(B) is formed as shown for the TiO_2_-3 material. Many of the diffraction peaks are typical for TiO_2_(B) while the remaining peaks belong to anatase TiO_2_ using the Powder Diffraction File (PDF) cards: TiO_2_ anatase, 00-21-1272; and TiO_2_(B), 00-046-1238. The peaks for TiO_2_(B) seen in TiO_2_-3 include diffraction planes due to (001), (110), (002), ($\bar{4}$01), (111), (310), (401), (003), ($\bar{6}$01), ($\bar{4}$21), ($\bar{1}$14), ($\bar{4}$23), and (621). In addition, the anatase TiO_2_ peaks are due to diffraction planes of (101), (103), (104), (112), (200), (105), and (211). It should be noted that the diffraction plane (001) of TiO_2_(B) and anatase TiO_2_ (101) overlap at approximately 2θ = 25°. The TiO_2_-3 material was prepared using a molar ratio of 0.60 for TiO_2_/SiO_2_ whereas the molar ratio of Ti(OC_2_H_5_)_4_/HCl was kept to be 0.74. Previous research using a different silica host, KIT-6 with a TiO_2_/SiO_2_ molar ratio of 0.68, led to the formation of TiO_2_(B) phase in conjunction with anatase TiO_2_ using either titanium isopropoxide or titanium butoxide as precursors for titania [32]. The molar ratio of Ti(OCH(CH_3_)_2_)_4_/HCl was maintained at 0.10 by Zhou and coworkers in their study.

**Figure 2:**
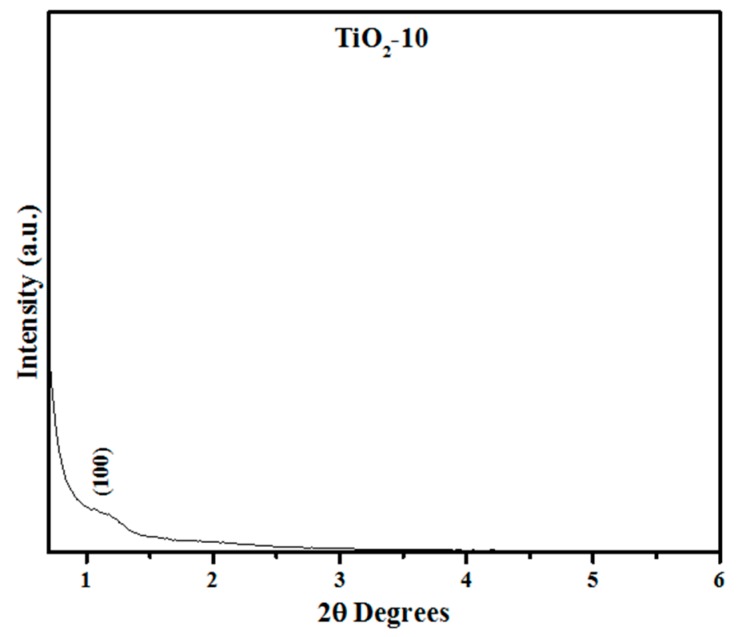
Powder X-ray diffraction of hard templated mesoporous TiO_2_ as function of impregnation cycles.

An increase in the number of cycles from three to eight leads to the formation of anatase-rutile mixed phases (TiO_2_-8) material as shown in Figure 2. The diffraction planes recorded for anatase phase in the TiO_2_-8 material include (101), (104), (112), and (200). The peaks from the rutile TiO_2_ phase include (110), (101), (111), (210), (211), (220), (004), (301), (112), and (320) diffraction planes. The Reference Intensity Ratio (RIR) method was used to calculate the percentages of various phases that are indicated in Table 1. The PDF card used for indexing the rutile TiO_2_ phase was 00-21-1276. Kim and coworkers reported the formation of anatase and rutile phases at similar ratios as ours [29,30]. Correspondingly, lower acidity conditions favor forming an anatase-rutile mixed phase TiO_2_ material [38] and the results obtained in this work are consistent with previous reports in literature. The additional impregnation cycles led to the formation of a minor amount of TiO_2_(B) in TiO_2_-10. The predominant peaks for TiO_2_(B) and anatase TiO_2_ are evident in Figure 1 for the TiO_2_-10 material. The TiO_2_(B) diffraction planes recorded for the TiO_2_-10 include (001), (110), (002), (111), (401), and (003). The anatase TiO_2_ phase peaks observed were due to diffraction planes of (101), (103), (104), (112), (200), (105), (211), (204), (116), (220), and (301). The number of peaks seen for the anatase TiO_2_ phase suggests that the TiO_2_-10 material is predominantly anatase, which is supported in RIR percentages in Table 1.

The change of the titanium alkoxide precursor from titanium ethoxide to titanium isopropoxide for impregnating SBA-15 is evident with the TiO_2_-4 material in Figure 1. This material has only anatase TiO_2_ phase. The diffraction planes recorded for anatase TiO_2_ phase include (101), (103), (104, (112), (200), (105), (211), (116), (220), and (301). Previous research suggests that use of titanium isopropoxide in nanocasting using periodic silica host materials leads to anatase TiO_2_ phase, similar to previous results [29,30,32]. Zhou and coworkers discovered that low calcination temperature of 300 °C led to anatase and TiO_2_(B) phases [32]. In summary, the powder XRD results shown in Figure 1 and Table 1, collectively, suggest that the loading and nature of the titanium alkoxide precursor affects the phase(s) formed.

In addition, short range powder XRD was completed for the TiO_2_-10 material as shown in Figure 2. The short range XRD plot indicate a small peak at 2θ near 1.25° due to (100) plane, suggesting some pore ordering. This is supported later in the discussion related to Transmission Electron Microscopy (TEM) results in Section 2.1.4. The short range powder XRD data of the remaining materials (TiO_2_-8 TiO_2_-3, and TiO_2_-4) are shown in the Supplementary Materials in Figures S1–S3. The results indicate that the periodic arrangement of the pores is destroyed in these materials. Thus, the results suggest that a certain amount of titania loading is required for retention of pores. At an optimal loading amount, the titania particles provide a robust environment for retention of pores. When the loading of titania is relatively low, they do not coat the pore walls of SBA-15 completely and hence on calcination, the pores collapse.

**Table 1:** Physical properties of mesoporous hard templated TiO_2_.

Material	Phase(s) (%) ^a^			Crystallite Size (s) (Å) ^b^			Bandgap (eV) ^c^
	Anatase	TiO_2_(B)	Rutile	Anatase	TiO_2_(B)	Rutile	
TiO_2_-3	48	52	-	68	17	-	3.35
TiO_2_-8	75	-	25	78	-	101	3.02
TiO_2_-10	98	2	-	104	207	-	3.18
TiO_2_-4	100	-	-	93	-	-	3.20

^a^ The percentages of three TiO_2_ phases (anatase, TiO_2_(B), and rutile) were calculated using the Reference Intensity Ratio (RIR) method provided in the Integrated Rigaku PDXL software version 2. In the RIR method, RIR values obtained from the Powder Diffraction File (PDF) files and the intensity of the peaks corresponding to the various phases are used to quantify the percentages of the phases; ^b^ Crystallite sizes of the mesoporous materials were calculated using the Halder-Wagner method. In the Halder-Wagner (HW) plot, y = (β/tanθ)^2^ is plotted against x = β/(tanθsinθ). The slope of the resulting straight line gives Kλ/D from which the crystallite size D can be calculated. K is a shape factor (dimensionless), λ = wavelength of Cu K_α_ radiation, and β = peak area/peak intensity. To be consistent, a standard set of peaks that do not overlap was used. The anatase peaks chosen are: 2θ = 25° (d_101_), 48° (d_200_), and 62.8° (d_204_). The TiO_2_(B) peaks chosen are: 2θ = 14° (d_001_), 25° (d_110_), 29.85° (d_111_), and 38° (d_401_). The rutile peaks chosen are: 2θ = 27° (d_110_), 36° (d_101_), and 41° (d_111_); ^c^ The bandgap of the mesoporous materials were estimated by extrapolation of the high slope region from the Kubelka-Munk plot (obtained from DRS studies) to the X-axis.

#### 2.1.2. N_2_ Physisorption

**Figure 3:**
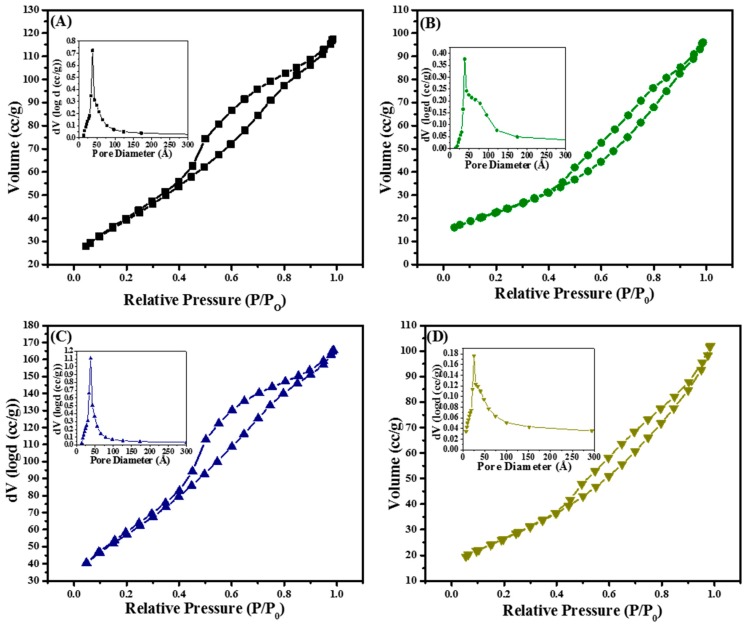
Nitrogen physisorption isotherms and Pore Size Distributions (insets) of hard templated mesoporous TiO_2_ (**A**) TiO_2_-10; (**B**) TiO_2_-8; (**C**) TiO_2_-3; and (**D**) TiO_2_-4 as function of cycles and titanium precursor.

The textural properties of the nanocasted titania materials were evaluated, and the isotherms with their respective Pore Size Distributions (PSDs) are shown in Figure 3A–D. The isotherms are typical type IV characteristic of mesoporous materials with hysteresis loops [52,53]. Figure 3A shows the nitrogen isotherm and the inset shows the PSD of TiO_2_-10 material. The N_2_ isotherm for TiO_2_-10 exhibits monolayer adsorption at low relative pressures (P/P_0_) of 0.05–0.30 that form the BET region. Following the BET section, an increase in relative pressure leads to formation of multilayers of nitrogen molecules with capillary condensation occurring on increasing the pressure to higher values. Hysteresis loop occur due to differences in the amount of nitrogen adsorbed during adsorption and desorption, and this is seen in the four materials in Figure 3. TiO_2_-10 material has H3 type hysteresis loop characteristic of materials having plate-like particle structure with pores of slit-like geometry. The TiO_2_-10 material does not exhibit a leveling off at the saturation vapor pressure at higher relative pressures, which suggests the pore structure is hierarchical in nature. The specific surface area is relatively high with 146 m^2^/g, but the pore volume is low (0.18 cm^3^/g) for the TiO_2_-10 material. The average PSD for the TiO_2_-10 is 50 Å as indicated in Figure 3A (inset), and this suggests a fairly uniform slit-like pore structure. On decreasing the number of impregnation cycles, the specific surface area reduces from 146 m^2^/g for TiO_2_-10 to 83 m^2^/g in the TiO_2_-8 material. Figure 3B shows the type IV isotherm for the TiO_2_-8 material similar to the TiO_2_-10. However, the PSD (inset in Figure 3B) shows a broader distribution of pores with average pore diameter of 72 Å. The TiO_2_-8 material has a H3 hysteresis loop typical of materials with slit-like pores. The PSD suggests a hierarchical arrangement of pores in the TiO_2_-8 material. Further decrease in impregnation cycles of Ti(OC_2_H_5_)_4_ leads to an increase in specific surface area (212 m^2^/g) and pore volume (0.26 cm^3^/g) for the TiO_2_-3 material as shown in Figure 3C. The TiO_2_-3 material also has type IV isotherm and H3 hysteresis loop is denoted by not leveling off at higher relative pressures corresponding to the saturation vapor pressure. The narrower PSD suggests a greater uniformity of pores for the TiO_2_-3 material (Figure 3C inset) compared to TiO_2_-10 and TiO_2_-8 prepared using titanium ethoxide as the precursor. The final material studied was TiO_2_-4 nanocasted using titanium isopropoxide with four impregnation cycles. The isotherm is shown in Figure 3D for the TiO_2_-4 material, which is again type IV characteristic of mesoporous materials. The inset of Figure 3D show the PSD for the TiO_2_-4 material, and the arrangement of pores for TiO_2_-4 material is hierarchical in nature similar to the TiO_2_-8 material. The specific surface area (97 m^2^/g) and pore volume (0.16 cm^3^/g) for the TiO_2_-4 material are in a similar range as the TiO_2_-8 material. In addition, the average pore diameter is close to 65 Å.

Overall, the nitrogen physisorption results indicate that the textural properties are dependent on the impregnation cycle. Table 2 shows the textural properties of the four nanocasted mesoporous titania materials, which are in a similar range as previous reports [29,30,34,35,37,38,40].

**Table 2:** Physico-chemical properties of mesoporous hard template TiO_2_.

Material	Specific Surface Area (m^2^/g) ^a^	Pore Volume (cc/g) ^b^	Average Pore Diameter (Å)
TiO_2_-10	146	0.18	50
TiO_2_-8	83	0.15	72
TiO_2_-3	212	0.26	48
TiO_2_-4	97	0.16	65

^a^ Specific surface area was determined by applying the Brunauer–Emmett–Teller (BET) equation to the relative pressure range of P/P_0_ = 0.05–0.30; ^b^ The pore volume was determined from the amount of N_2_ adsorbed at the highest relative pressure (P/P_0_) of approximately 0.99.

#### 2.1.3. Diffuse Reflectance Spectroscopy

**Figure 4:**
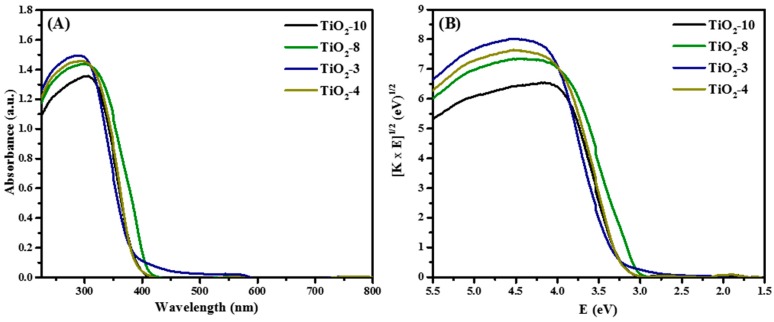
DRS of hard templated TiO_2_ mesoporous materials as function of cycle and titanium precursor (**A**) Absorbance plot and (**B**) Tauc plot.

The optical response of the mesoporous titanium dioxide materials was evaluated and the Diffuse Reflectance (DR) spectra are shown in Figure 4.

Diffuse reflectance spectroscopy was completed to determine the effects of the various phases on the bandgap. Figure 4A shows the absorbance plot. The corresponding Tauc plot is shown in Figure 4B. The strong absorption in the UV region (< 400 nm) are due to the optical band gap of TiO_2_, which typically lie in this region. The smallest bandgap was obtained for TiO_2_-8 material having anatase-rutile mixture, and this is consistent from the smaller bandgap of approximately 3.0 eV for rutile. The TiO_2_-10 and TiO_2_-4 had similar bandgap energies because they are composed of mainly anatase or exclusively anatase, with bandgaps of close to 3.2 eV. Greater amounts of TiO_2_(B) in TiO_2_-3 material with small crystallite size led to a larger bandgap of 3.35 eV. In addition, the crystallite sizes are consistent with the bandgap energies obtained, as shown in Table 1.

#### 2.1.4. Transmission Electron Microscopy

**Figure 5:**
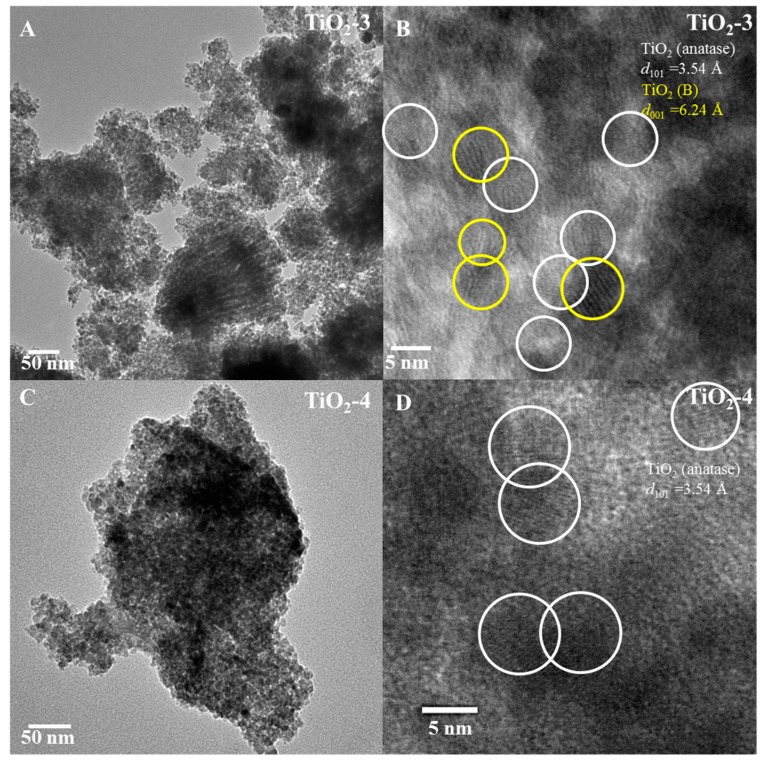
TEM images. TiO_2_-3 ((**A**) low magnification and (**B**) high magnification) and TiO_2_-4 ((**C**) low magnification and (**D**) high magnification) nanocasted titania mesoporous materials. The yellow and white circles indicate TiO_2_(B) and anatase phase respectively.

Transmission electron microscopy (TEM) studied were completed to evaluate the morphology and phase(s) present in these materials. The low magnification TEM image of TiO_2_-3 shown in Figure 5A indicates that this material has irregular aggregates of particles. High magnification TEM analysis of TiO_2_-3 (Figure 5B), shows that lattice fringes are present for anatase TiO_2_ and TiO_2_(B), and the diffraction planes in the lattice fringes are from anatase (101) and (001) from TiO_2_(B) with heterojunctions forming between these two phases. Figure 5C shows the low magnification of TiO_2_-4, and the high magnification of this material is indicated in Figure 5D. Figure 5D shows the lattice fringes from the crystallographic (101) plane of anatase.

**Figure 6:**
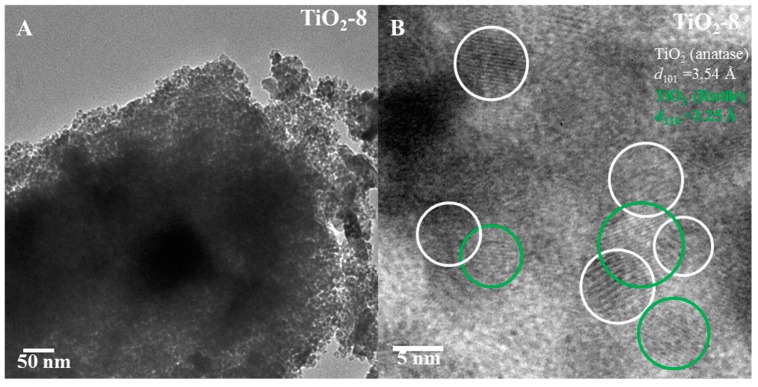
TEM images of TiO_2_-8 ((**A**) low magnification and (**B**) high magnification) nanocasted titania mesoporous material. The green and white circles denote rutile and anatase phase respectively.

Figure 6A,B shows the TEM images of TiO_2_-8 material. The pore arrangement is random as indicated in Figure 6A. High magnification image indicates lattice fringes due to anatase (101) and rutile (110) with some heterojunction formation between the anatase and rutile phases.

**Figure 7:**
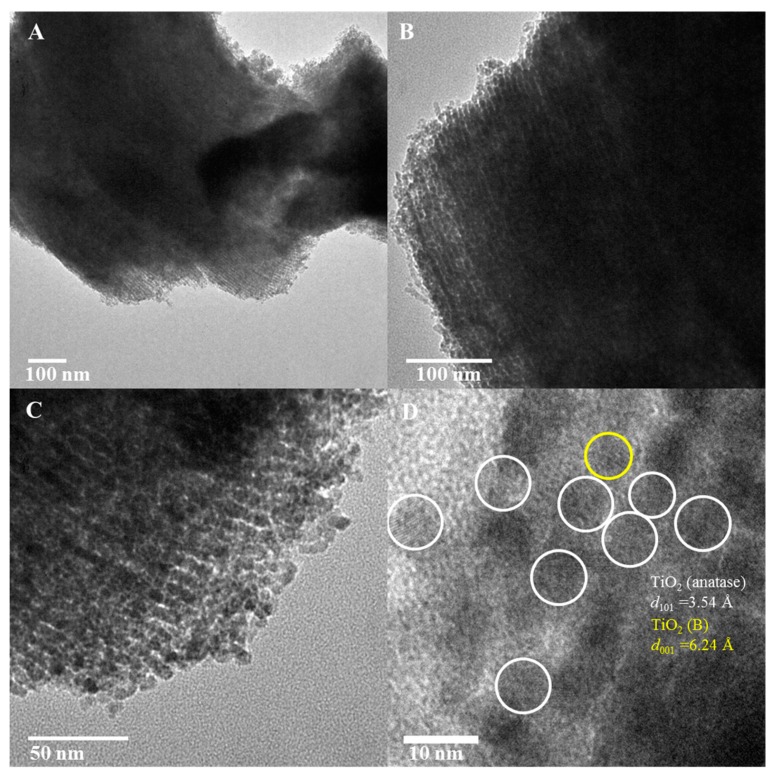
TEM of TiO_2_-10 ((**A–C**) low magnification and (**D**) high magnification) nanocasted titania mesoporous materials. The yellow and white circles represent TiO_2_(B) and anatase phase respectively.

The TEM images in Figure 7A–C show the unidirectional pore channels present in TiO_2_-10 material. Long unidirectional pore channels, similar to the host periodic mesoporous silica SBA-15 material, are seen in these images. The low magnification TEM results for TiO_2_-10 are consistent with low-angle powder XRD of this material in Figure 2. An over-focus image of TiO_2_-10 is shown in Figure 7D. Lattice fringes due to (101) and (001) diffraction planes of anatase and TiO_2_(B) respectively are observed. Overall, the TEM analysis supports powder XRD results with heterojunctions formed for the three mesoporous materials prepared using titanium ethoxide, and only pure anatase TiO_2_ phase in TiO_2_-4 made from titanium isopropoxide.

#### 2.1.5. Scanning Electron Microscopy

**Figure 8:**
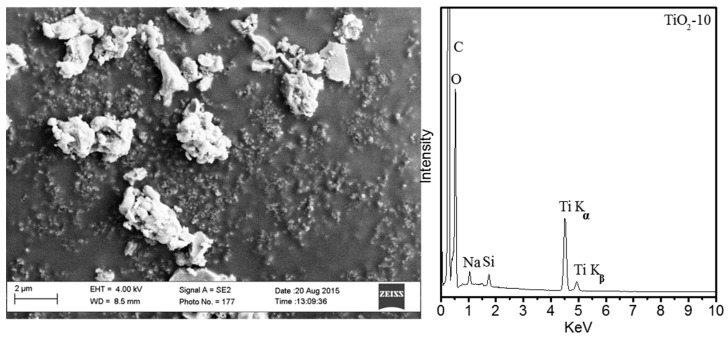
SEM image of TiO_2_-10 hard templated-TiO_2_ mesoporous material and EDX mapping of this material.

Figure 8 shows the scanning electron microscopy image of TiO_2_-10 with accompanying Energy Dispersive X-ray (EDX) mapping of this sample. The SEM image shows irregular morphology for the TiO_2_-10 material, and EDX mapping suggests a small amount of silica from the SBA-15 template after removing with NaOH aqueous solution. The retention of a small amount of silica is fairly common using the nanocasting method. Overall, the TEM and SEM analyses suggest TiO_2_-10 periodic unidirectional pore channels with globular morphology. The SEM of TiO_2_-3, -4, and -8 are shown in Figures S4–S6.

### 2.2. Solar Hydrogen Evolution

**Figure 9:**
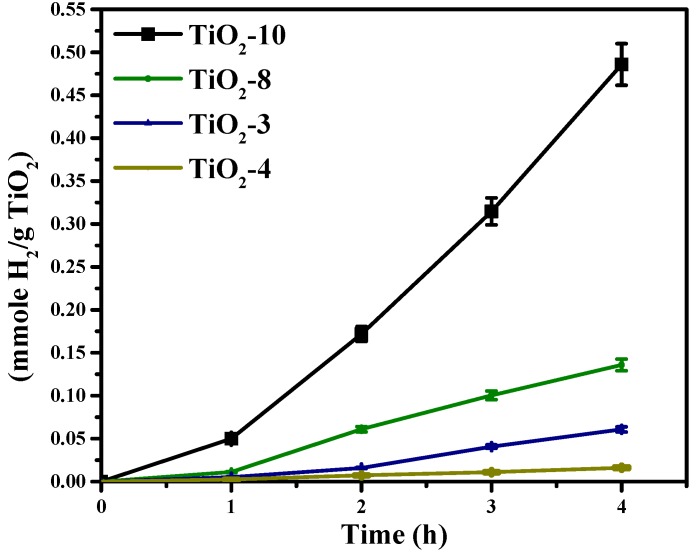
Hydrogen evolution from the hard templated mesoporous TiO_2_ photocatalysts.

The photocatalytic activity for solar hydrogen production was evaluated for the nanocasted titania mesoporous materials and are shown in Figure 9. The photocatalytic activity was noted to be in the following order of TiO_2_-10 > TiO_2_-8 > TiO_2_-3 > TiO_2_-4. The materials made with titanium ethoxide had mixed phases, and the TiO_2_-4 material synthesized using titanium isopropoxide had only anatase phase. Therefore, the mixed phases may impart enhanced charge separation resulting in increased photocatalytic activity. Within the materials prepared using titanium ethoxide, TiO_2_-3 had 48% anatase and 52% TiO_2_(B) and TiO_2_-8 was found to have 75% anatase and 25% rutile content as indicated in Table 1. The TiO_2_-10 material was noted to have 98% anatase TiO_2_ and 2% TiO_2_(B).

The photocatalytic activity of titania is a function of several factors, such as crystallinity, crystallite size, phase(s), surface area, porosity, *etc.* The crystallinity trend is TiO_2_-10 ≈ TiO_2_-4 > TiO_2_-8 > TiO_2_-4. However, crystallinity alone may not be the only factor responsible for the relative high activity of TiO_2_-10. This is because TiO_2_-10 produces much greater hydrogen than TiO_2_-4. TiO_2_-8 and TiO_2_-3 have similar photocatalytic activity, so differences in crystallinity may not fully explain the trends obtained in production of hydrogen. The crystallite sizes vary, as shown in Table 1. However, TiO_2_-10 and TiO_2_-4 anatase crystallite sizes vary little, but the TiO_2_(B) crystallite size significantly varies for TiO_2_-10 and TiO_2_-3. Therefore, the crystallite size and crystallinity appear to have a modest influence on the photocatalytic activity. Table 2 shows the porosity of these four materials, and the specific surface area is the highest for TiO_2_-3. If specific surface area was an important factor, then, the solar hydrogen evolution should be the largest for TiO_2_-3. However, this is not the case. Furthermore, the average pore diameter for TiO_2_-10 and TiO_2_-3 are similar, so the porosity (specific surface area and pore size) does not seem to be a major factor. As illustrated in the TEM images (Figure 7A–D), the periodic arrangement of pores seems to have been retained in TiO_2_-10 in comparison to the rest of the three mesoporous materials. The photocatalytic activity of TiO_2_-10 was found to be the highest in this study. Previously, we have observed that the pore architecture is an important factor for hydrogen evolution [54]. We reported that the photocatalytic activity was higher when a periodic mesoporous silica support was used to disperse titania and Pd nanoparticles in comparison to an aperiodic mesoporous silica support. The results from this study also indicate that the presence of such periodic pores (albeit not highly uniform), leads to effective hydrogen evolution. In addition to the critical role of the support, the phase(s) formed and their relative percentages may also play a role as well. The TiO_2_-10 material has perhaps an optimal amount of anatase and TiO_2_(B), which minimizes electron-hole recombination. The present outcome is consistent with prior observations with mixed phase titania for solar hydrogen evolution [43]. In order to understand this better, photoluminescence studies were carried out.

### 2.3. Photoluminescence

**Figure 10:**
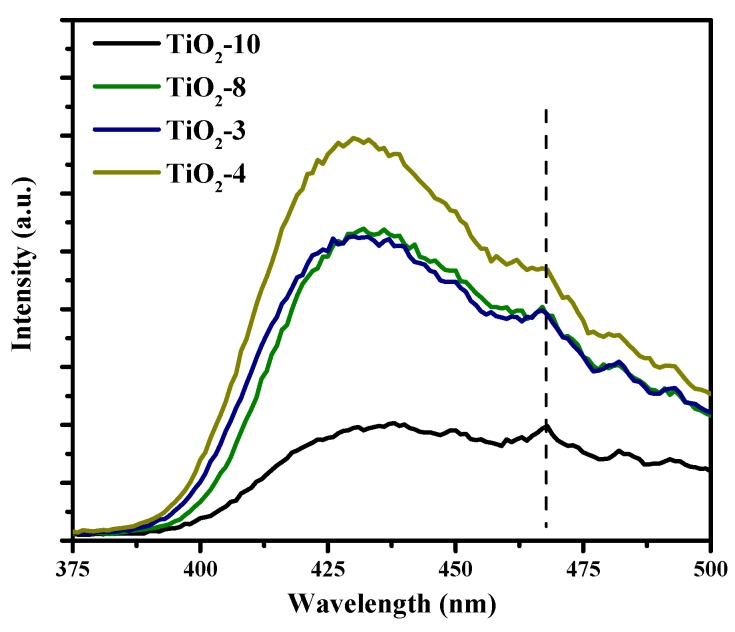
Photoluminescence of hard templated mesoporous TiO_2_.

The phase(s) of titania appear to play a role in the solar hydrogen production photocatalytic activity. We endeavored to further understand the trends in photocatalytic hydrogen evolution. Therefore, photoluminescence studies were conducted on the four mesoporous materials as shown in Figure 10. All the samples show an intense peak that appears near 430 nm that is attributed to electron-hole recombination due to self-trapped excitons from all phase(s) of titania. In addition, small peaks can be seen near 467, 482, and 492 nm. The presence of oxygen defects can be inferred from the small peaks seen at approximately 467 nm, whereas the peaks at 482 and 492 nm may be attributed to surface states. These trap states will facilitate trapping charge carriers. The PL results supports the trends in photocatalytic activity previously shown in Figure 9. TiO_2_-4 material comprised solely of anatase and has the largest photoluminescence from increased electron-hole recombination, and correspondingly, the amount of hydrogen produced from this material is the lowest. The photoluminescence from TiO_2_-8 and TiO_2_-3 are similar. In comparing the photocatalytic activities, one notices that the amount of hydrogen produced is similar after 1 h of irradiation. On irradiation for longer durations of time, some difference is observed in the yield of hydrogen in these two materials. The photoluminescence signal from TiO_2_-10 was significantly lower in comparison to the rest of the mesoporous materials, and this suggests significantly reduced electron-hole recombination. This is consistent with the relatively high photocatalytic activity obtained for this material. In summary, the photoluminescence studies validate the trends observed in photocatalytic production of hydrogen.

## 3. Materials and Methods

### 3.1. Materials

Titanium ethoxide, Ti(OC_2_H_5_)_4_, (Acros Organics, Pittsburg, PA, USA), titanium isopropoxide, Ti(OCH(CH_3_)_2_)_4_, (Acros Organics, 98+%), conc. HCl (Fisher-Scientific, ACS grade, 37 wt %), and sodium hydroxide, NaOH, (Acros Organics, analysis grade) were used for the synthesis of the titanium dioxide. Deionized water of 18.2 MΩ cm water was employed throughout the synthesis.

### 3.2. Synthesis

The hard template synthetic or nanocasting method was employed for preparing mesoporous titanium dioxide materials [30]. In a typical hard template synthesis, various microliters of titanium ethoxide or titanium isopropoxide were added to a known amount of deionized (DI) water. This solution was stirred for 30 min to form an amorphous titania precipitate. The total amount of titanium precursor added was dependent on the number of cycles completed. After the initial step of forming the amorphous titania precipitate, the aqueous suspension was centrifuged for 30 min, and the white solid was dissolved in concentrated HCl. This solution was added to the periodic silica SBA-15 host under stirring for 30 min. Then, the mixture was heated to 160 °C for 10 min, which concluded 1 cycle. These steps were repeated to obtain the desired number of impregnation cycles. Finally, the mixture was heated for 24 h at 100 °C and then ground to a fine powder. The dry and finely grounded composite was subjected to calcination at 450 °C for 3 h at a heating rate of 3 °C/minute. The final step involved etching the calcined composite with 17 mL of 1M NaOH for 12 h under stirring followed by centrifugation and washing with deionized water resulting in a white material.

The periodic SBA-15 material was prepared according to a previous report published by us [55].

### 3.3. Characterization and Photocatalytic Studies

The hard template TiO_2_ mesoporous materials were extensively characterized by many techniques. Powder X-ray diffraction analysis of the mesostructured materials were recorded under ambient conditions using a Rigaku Ultima IV instrument (Akishima-shi, Tokyo, Japan) with Cu Kα radiation (λ = 1.5408 Å). The accelerating voltage used was 40 kV, and the emission current employed was 44 mA. In addition, the powder XRD analysis was completed by scanning from 2θ = 10° to 80° for evaluating the phase and crystallinity of the titania materials. The materials were scanned at 0.02° step width and the scan rate was 1°/min. The samples were scanned from 2θ = 0.6° to 6° for determining the periodic nature. The percentages of various phases of TiO_2_ were determined using Reference Intensity Ratio (RIR) method with the Rigaku PDXL version 2 software. The textural properties such as specific surface area, pore volume, and pore diameter distribution, were delineated with N_2_ physisorption measurements. The materials were dried overnight at 80 °C and extensively degassed at 110 °C for 7.5 h. The N_2_ isotherms were collected at 77 K (−196 °C, liquid nitrogen temperature) using at NOVA 2200*e* (Quantachrome Instruments) surface area and pore volume analyzer. The specific surface areas were calculated using the Brunauer–Emmett–Teller (BET) equation from the relative pressure range (P/P_0_) of 0.05–30. The pore volumes were determined from the amount of nitrogen adsorbed at the highest relative pressure (P/P_0_) of ~0.99. The average pore diameter was calculated using the equation, average pore diameter = 4 (pore volume)/(specific surface area). The UV-Vis diffuse reflectance spectra were completed using a Cary 5000 UV-Visible spectrophotometer (Santa Clara CA, USA) equipped with a Harrick Scientific praying mantis accessory. The bandgaps of the samples were evaluated by extrapolating the highest slope region to the X-axis in the Tauc plot, and this was completed by transforming the absorbance plot using the Kubelka–Munk function. Transmission electron microscopy (TEM) images were recorded using a Tecnai G^2^ instrument (Hillsboro, OR, USA) operating at 120 kV. Before the TEM analysis, the TiO_2_ samples were dispersed in ethanol, and the suspensions were subjected to centrifugation for 30 min. Then, one drop of the suspension was placed onto the copper grid coated with carbon film followed by overnight drying under ambient conditions. Scanning electron microscopy (SEM) analysis was completed using Zeiss Sigma instrument. An Oxford X-max detector was used for EDX mapping. The acceleration voltage used was 12 kV. Photoluminescence measurements were completed with a Horiba Jobin Yvon-Fluoromax 4 instrument (Edison, NJ, USA). The excitation wavelength was 275 nm, and the emission spectra were monitored in the range of 375 to 500 nm.

The photocatalytic water splitting experiments were completed as follows. A known ratio of 1 g of photocatalyst per Liter of solution was placed in the photoreactor, and suspension contained water-methanol mixture with molar ratio of [H_2_O]/[CH_3_OH] = 8. The resulting mixture was degassed with ultrahigh purity Ar before irradiation. The suspensions were stirred extensively over the course of the experiment. A 300 W Xe Oriel lamp with appropriate filter (Oriel Filter 57396, Franklin, MA, USA) was employed as the UV light source. The concentration of hydrogen was determined by gas chromatography using a SRI 8610 C instrument (Torrance, CA, USA) that had a molecular sieve column coupled to a thermal conductivity detector (TCD). The amount of hydrogen formed was calculated from a calibration curve made previously.

## 4. Conclusions

A nanocasting method using silica SBA-15 as hard-template was explored for forming mesoporous titanium dioxide. This synthetic method afforded mixed phases of titania by simply varying the number of impregnation cycles and nature of the titanium alkoxide precursor. Depending on the experimental conditions, phases of anatase, rutile, or TiO_2_(B) phases were formed. The mesoporous material TiO_2_-10, formed after ten impregnation cycles, exhibited the highest photocatalytic activity. The periodic nature of the pores in the TiO_2_-10 material was an important factor that contributed to enhanced solar hydrogen production. The synthesis method provides an avenue for fabricating periodic mesoporous titanium dioxide for various other applications such as photocatalytic degradation of organics and dyes and dye-sensitized solar cells.

## Supplementary Materials

Supplementary materials can be accessed at: http://www.mdpi.com/1420-3049/20/12/19812/s1.
