# Supplementary material for: Nanocasting of Periodic Mesoporous Materials as an Effective Strategy to Prepare Mixed Phases of Titania
Source: Molecules. 2015 Dec 8;20(12):21881–95. doi: 10.3390/molecules201219812 (PMC6331994; doi:10.3390/molecules201219812)
Supplement: Supplementary file 1 [file molecules-20-19812-s001.pdf]

Supplementary Materials: Nanocasting of Periodic Mesoporous Materials as an Effective Strategy to Prepare Mixed Phases of Titania

Luther Mahoney, Shivatharsiny Rasalingam, Chia-Ming Wu and Ranjit T. Koodali

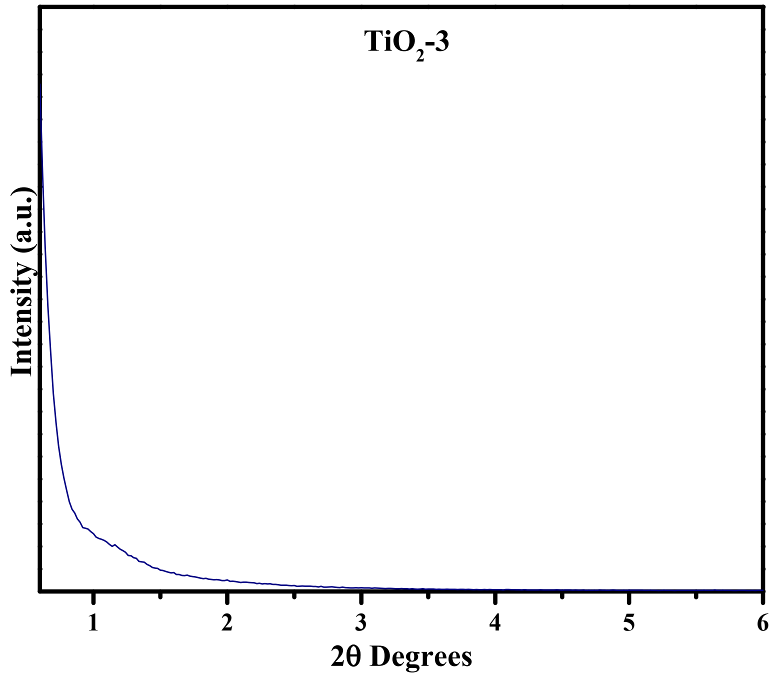

Figure S1. Low-angle powder X-ray diffraction of hard templated mesoporous TiO_2 using titanium ethoxide after three cycles of impregnation.

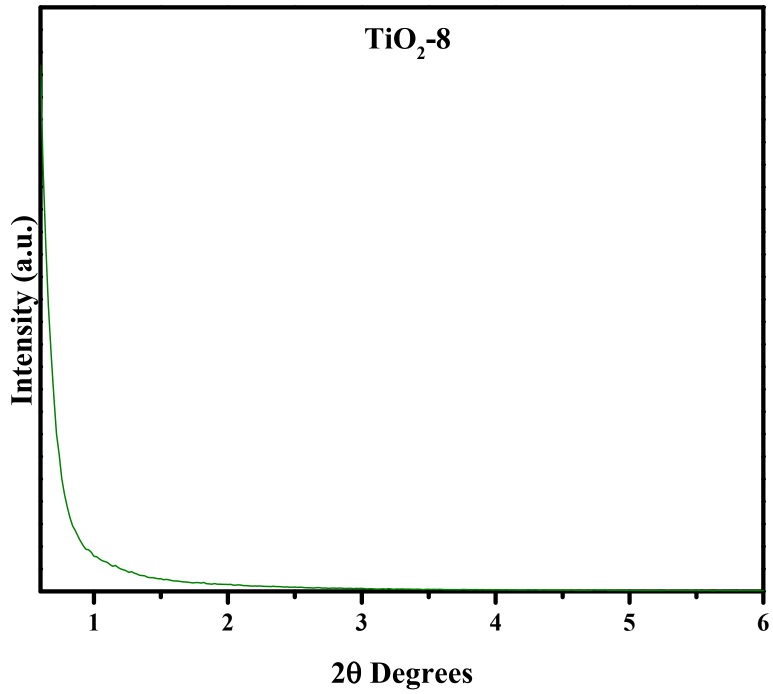

Figure S2. Low-angle powder X-ray diffraction of hard templated mesoporous TiO_2 using titanium ethoxide after eight cycles of impregnation.

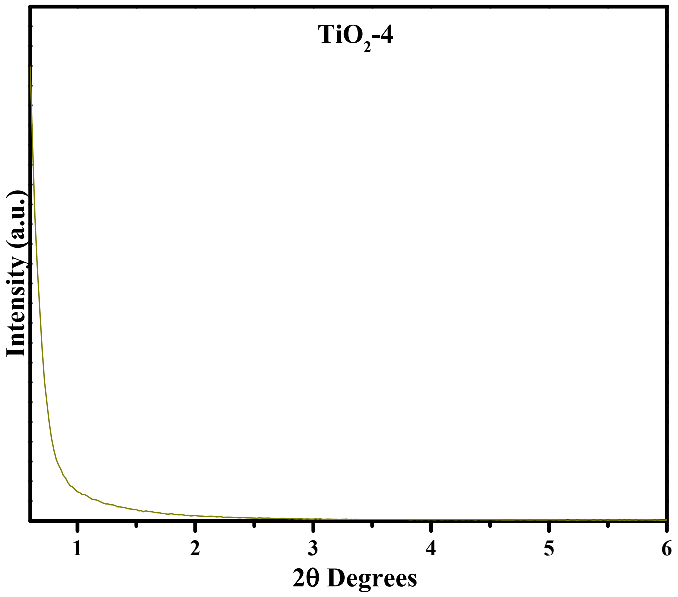

Figure S3. Low-angle powder X-ray diffraction of hard templated mesoporous TiO_2 using titanium ethoxide after four cycles of impregnation.

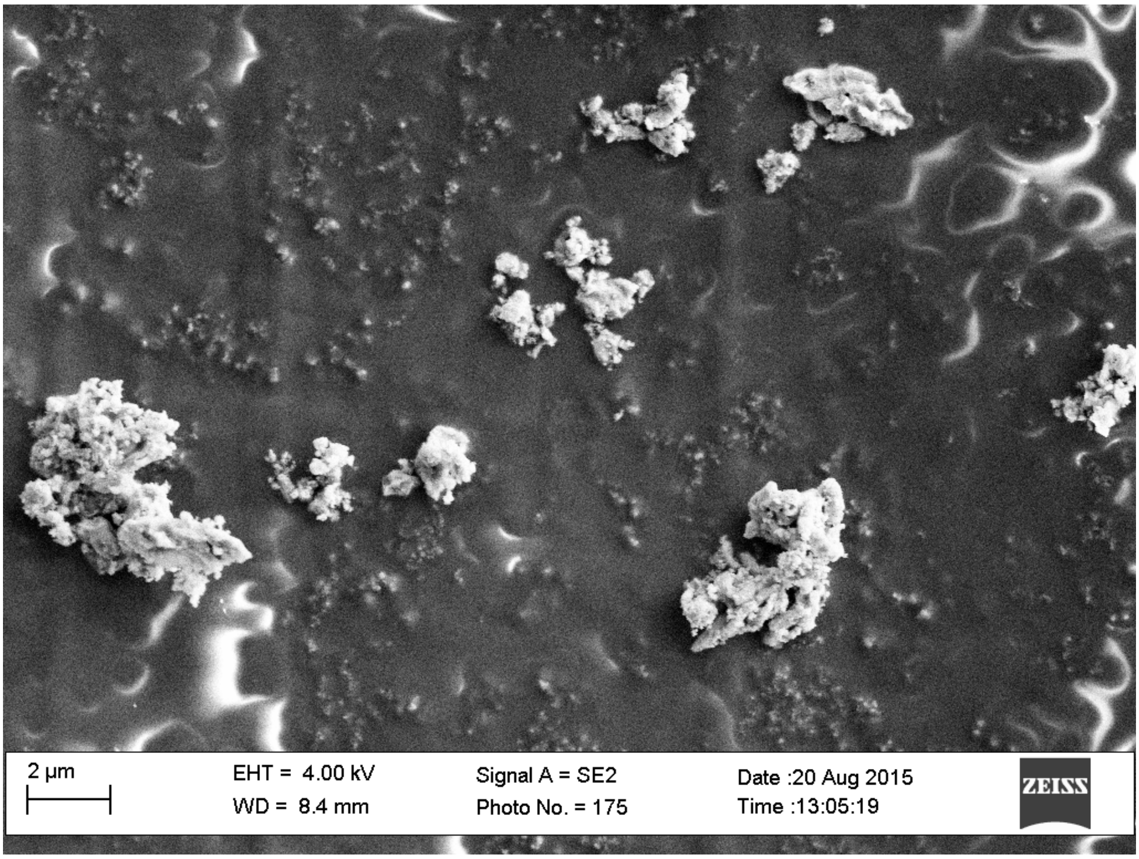

Figure S4. SEM image of TiO_2-3 hard templated-TiO_2 mesoporous material.

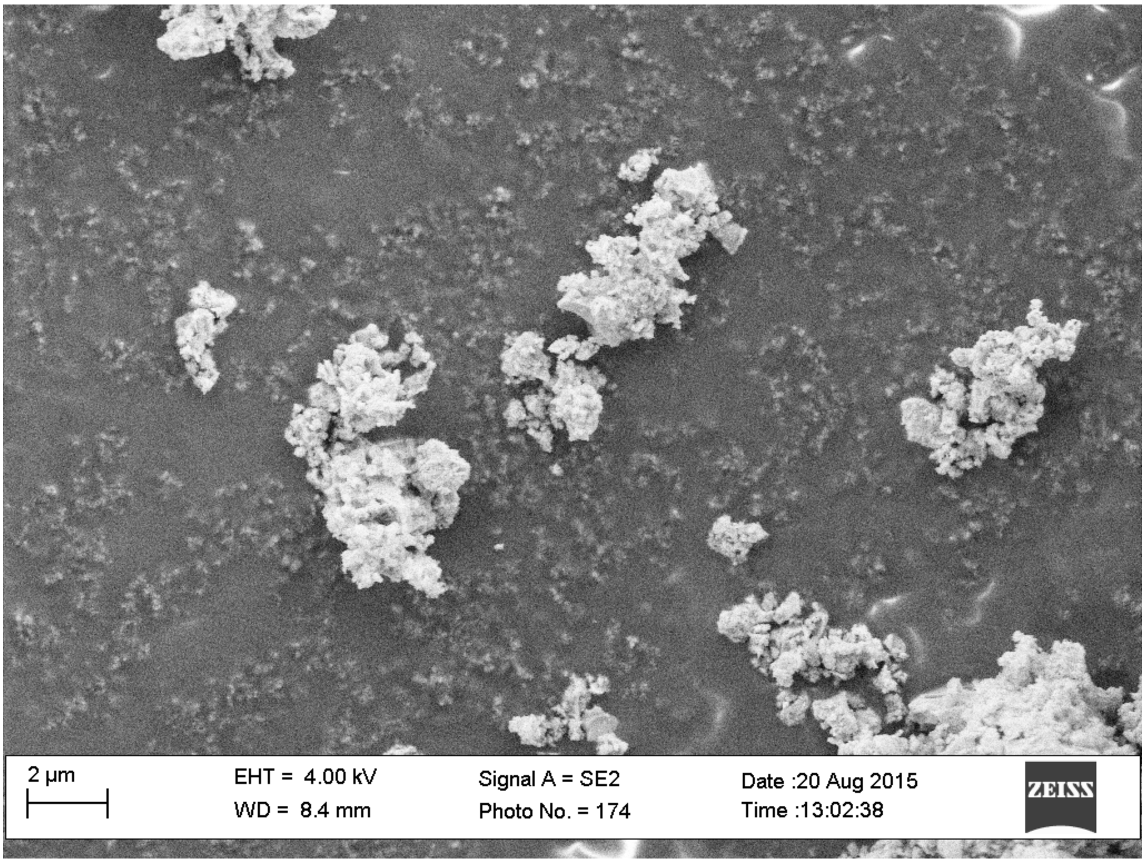

Figure S5. SEM image of TiO_{2-4} hard templated-TiO_2 mesoporous material.

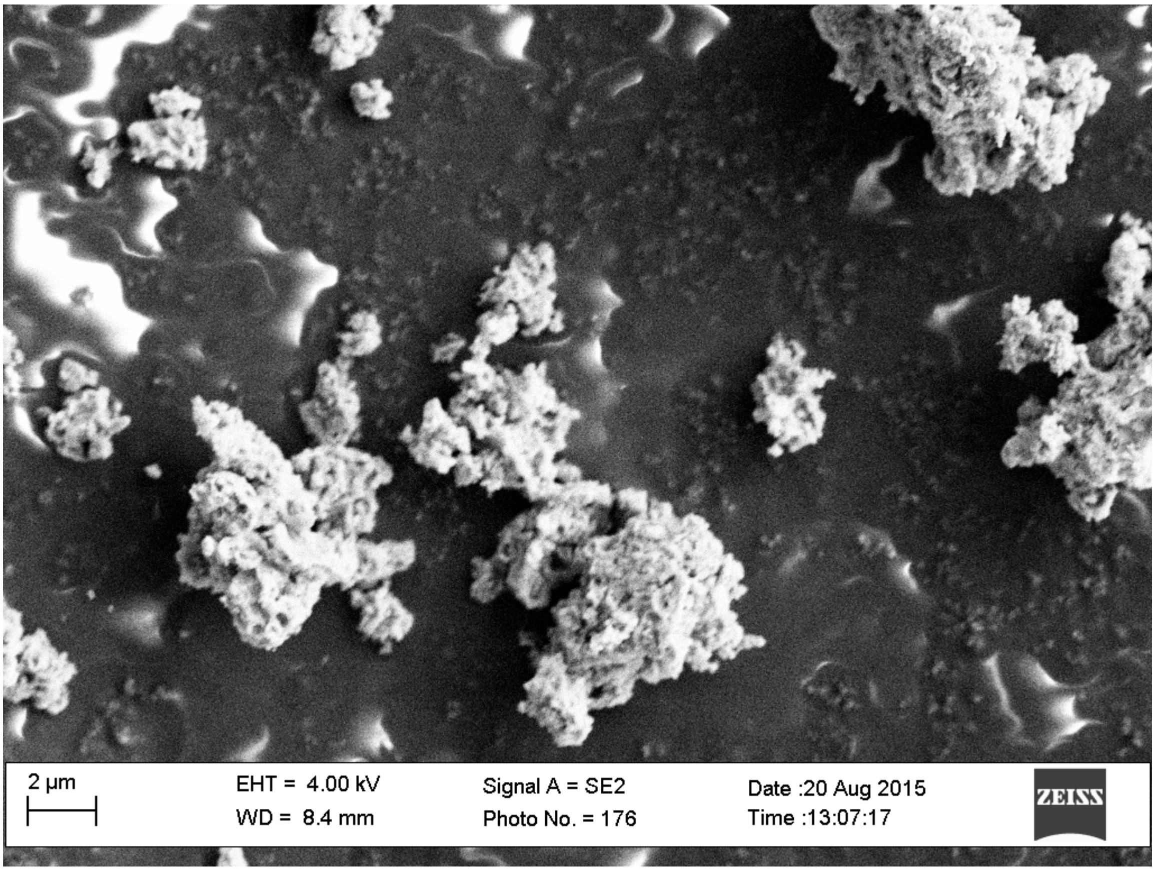

Figure S6. SEM image of TiO2-8 hard templated-TiO_2 mesoporous material.
